# A mixed-methods study on collaborative health governance for older adults with mild cognitive impairment in Hangzhou, China

**DOI:** 10.3389/fpubh.2025.1720145

**Published:** 2025-11-21

**Authors:** Yiqi Kong, Zihan Xu, Zhiyou Zhu, Lan Ye, Siyu Zhou

**Affiliations:** 1 School of Public Administration, Hangzhou Normal University, Hangzhou, China; 2 Party School of Shanghai Municipal Committee of the Communist Party of China, Shanghai, China

**Keywords:** mild cognitive impairment, Alzheimer’s disease, community-based management, optimization, collaborative health governance

## Abstract

**Introduction:**

Mild cognitive impairment (MCI), a prodromal stage of Alzheimer’s disease, poses a critical public health challenge in aging populations. Current community-based MCI interventions are often fragmented, lacking effective collaboration among families, community workers, and physicians. This study aimed to construct a multi-agent collaborative governance model and propose optimization strategies for community-based MCI management.

**Methods:**

A mixed-methods design was employed in Hangzhou, China. Quantitatively, 373 community-dwelling older adults with MCI completed the Family APGAR Index, Social Isolation Scale, and Mini-Mental State Examination (MMSE). Structural equation modeling (SEM) was used to analyze the pathways linking social isolation, family support, and cognitive domains. Qualitatively, thematic analysis was conducted on in-depth interviews with four community workers and four family physicians to delineate stakeholder responsibilities and collaboration challenges.

**Results:**

The quantitative results revealed a dual effect of family support: it was positively associated with memory and calculation ability (path coefficient = 0.176, *p* < 0.05) but negatively associated with reading and praxis ability (path coefficient = −0.164, *p* < 0.05). Reduced social isolation significantly enhanced family support (*β* = 0.405, *p* < 0.001). Furthermore, positive feedback loops were identified among cognitive domains (orientation, reading/praxis, memory/calculation). Qualitatively, key barriers included the absence of structured collaboration mechanisms, passive information-sharing, and insufficiently trained family caregivers.

**Discussion and conclusion:**

This study underscores the complex role of family support and the necessity of integrated care. We propose a tripartite “Social Worker–Physician–Family” collaborative framework, featuring skill-building curricula, psychological support, and cross-sectoral data linkage protocols to optimize health outcomes. However, due to the cross-sectional design, causal inferences are limited, and longitudinal studies are needed to validate the pathways. The findings offer empirical evidence and practical insights for designing community-based interventions for early-stage cognitive impairment in aging societies.

## 1 Introduction

Mild cognitive impairment (MCI), a prodromal stage between normal cognition and Alzheimer’s disease, is associated with an estimated conversion rate of up to 80% to dementia (1). Furthermore, epidemiological data indicate that 15.5% of Chinese adults aged ≥60 years have MCI (2), representing over 20% of global MCI and dementia cases in this age group. However, MCI interventions often focus on severe stage treatments by specialists in tertiary hospitals, including pharmacotherapy, traditional Chinese medicine (3), and cognitive training (4). This hospital-centric model neglects two critical elements: proactive health support from primary care providers and effective multistakeholder coordination. Consequently, intervention continuity becomes fragmented, and referral rates for screening-detected cases remain inadequate (5). Meanwhile, from the perspective of home care, there is a disconnect between family and society, with 49% of families misinterpreting MCI as natural aging (6). Social stigma leads to concealment of the condition and delays the expectation of timely intervention in some families (7). These challenges highlight the limitations of fragmented health interventions and underscore the urgent need to build an unimpeded collaborative health governance model involving multiple stakeholders.

Three primary models govern the health management of older adults with MCI in community settings: family based, community-driven, and multistakeholder collaborative models. In brief, the family based model utilizes the home environment as the core setting, where caregivers deliver non-pharmacological interventions that emphasize personalized integration into daily life, such as customized activity plans (8) and caregiver-participatory reminiscence therapy (9). The community-driven management model is primarily coordinated through community health centers, which provide integrated services including early detection, daily living support, and health education (10). In China, older adult care institutions have progressed into integral components of this model by merging medical and aged-care resources (11). The multistakeholder collaborative model emphasizes a government-steered framework that integrates families, community organizations, and care institutions to establish a seamless service network (12, 13). Complementing this, research has contributed to the gradual formation of a three-tiered screening consensus for community-based MCI management, encompassing self/family assessment, community health center evaluation, and specialist hospital referral (14, 15).

However, each of these models demonstrates significant constraints. The family based model is highly dependent on the health literacy of caregivers and often leads to increased caregiver burden (16). The community-driven model frequently engenders passive patient engagement coupled with insufficient disease awareness and a lack of specialized training for primary care physicians (17). The multistakeholder collaborative model contends with fundamental structural challenges, including service demand mismatches and inadequate collaborative governance mechanisms (18), which result in insufficient inter-stakeholder coordination and ultimate failure to address the heterogeneous health needs of patients with MCI. This gap is also evident internationally. Recent frameworks like the World Health Organization’s (WHO) Integrated Care for Older People (ICOPE) approach emphasize the need for coordinated, person-centered interventions that are not yet fully realized in community practice (19). Similarly, the Organisation for Economic Co-operation and Development (OECD) has highlighted the pressing need to strengthen primary and community care for dementia and its at-risk stages, like MCI, through better care coordination and multidisciplinary teamwork (20). Concurrently, the application of Artificial Intelligence (AI) for early screening of cognitive impairment in community settings is emerging as a promising tool to enhance detection efficiency and scalability, though its integration into routine care remains nascent (21). These international developments underscore a global consensus on the importance of integrated, community-based models for cognitive health, against which the fragmentation observed in our current system becomes even more apparent.

To address these systemic gaps, this study aimed to construct an analytical framework based on collaborative governance and social support theories. From the perspective of collaborative governance theory, health management for older adults with MCI involves multidimensional needs that cannot be adequately addressed by any single entity. Instead, it requires complementary resources and institutionalized collaboration among social workers, family physicians, and family members to overcome the inefficiencies inherent in the provision of fragmented services. From the perspective of social support theory, the synergy between family (informal) support and community (formal) support can help buffer the pressures associated with cognitive decline. Reducing the level of social isolation can alleviate family burdens by enriching external support networks, thereby facilitating a shift in family care from a survival-oriented to a development-oriented approach, and ultimately promoting improvement across all dimensions of cognitive function.

Therefore, this study employs a mixed-methods approach to: (1) quantitatively examine the pathways through which social isolation and family support influence specific cognitive domains among older adults with MCI, and (2) qualitatively explore the current state and challenges of multistakeholder collaboration from the perspectives of community workers and family physicians. The ultimate goal is to propose an optimized collaborative governance model and evidence-based strategies for community-based MCI management.

## 2 Methods

### 2.1 Hypothetical framework

This study is an empirical research article that employs a mixed-methods design to investigate the collaborative health governance for community-dwelling older adults with MCI. The extensive hypothesis system presented below is derived from the collaborative governance and social support theories mentioned in the Introduction, and serves as the analytical framework for our quantitative investigation.

**Figure 1:**
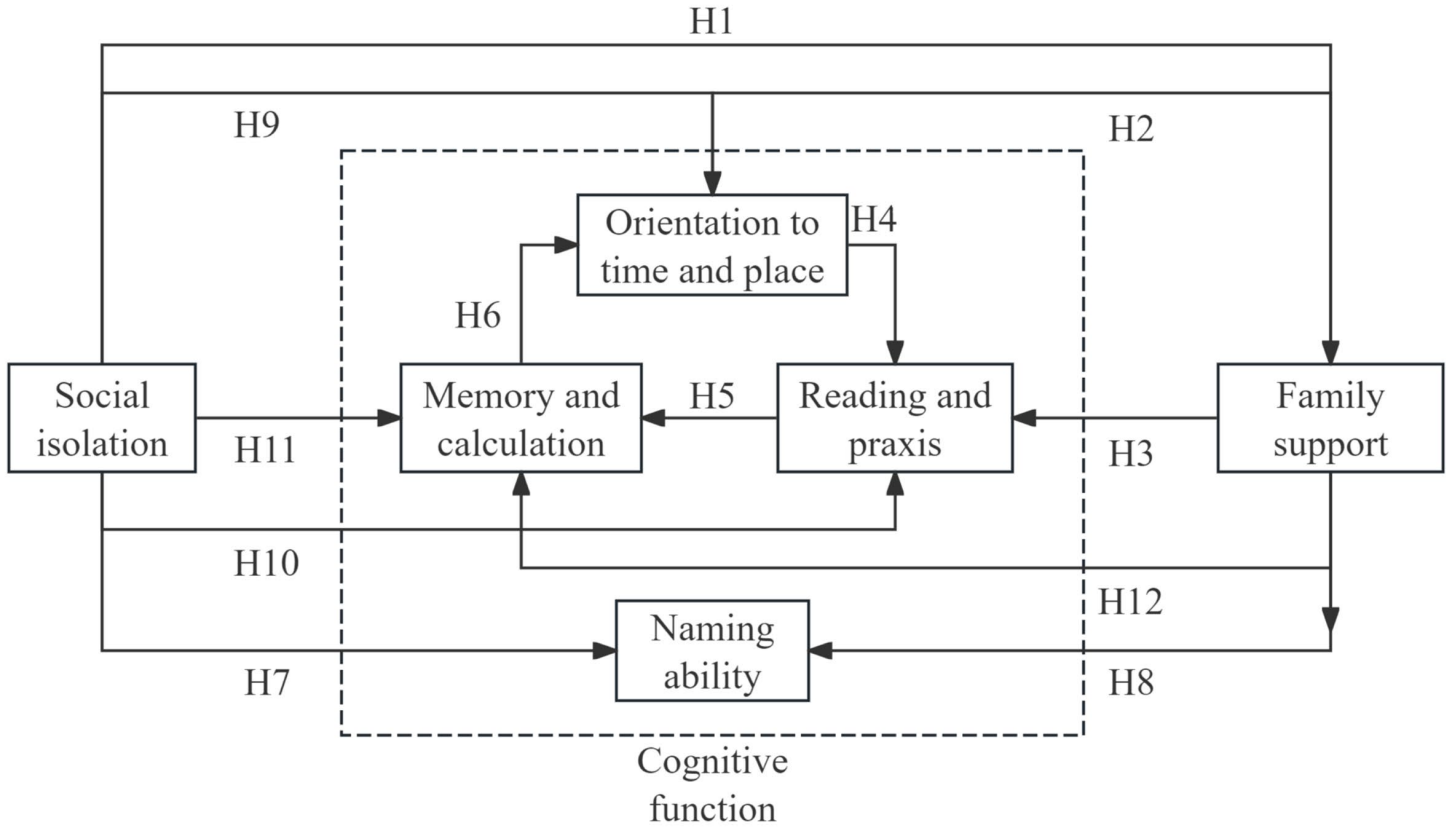
Research hypotheses.

Within the community context, this study focused on collaboration among multiple stakeholders. Building on the characteristics of existing multistakeholder collaboration models, we explored the pathways of collaborative governance from the perspective of three main service providers: family members, family physicians, and community workers. Based on this, the study proposes the following overarching hypothesis. Collaborative behavior among multiple stakeholders and social support networks jointly influence the cognitive levels of older adults with MCI. Optimizing the collaborative governance mechanism of multiple stakeholders will further promote positive health outcomes in this population. Guided by collaborative governance and social support theories, this study proposes the following 12 hypothetical pathways (Figure 1):

Hypothetical pathway 1 (*H1*): The level of social isolation negatively influences the level of family support. The family is a vital component of social support systems. Family support is a significant predictor of social isolation in older adults (22, 23). Higher levels of family support are associated with a lower risk of social isolation. Building on this, the present study explored the reverse effect: how the degree of social isolation may, in turn, diminish family support.

*H2*: Family support positively influences memory and calculation ability. The maintenance of memory and calculation ability relies on continuous cognitive stimulation. As the primary setting for interaction among older adults, the family provides diverse environmental stimuli, such as daily conversations and emotional support, which can promote neural plasticity (24, 25), thereby positively enhancing memory and calculation performance.

*H3*: Family support negatively influences older adults’ independent reading and praxis ability. Sustaining reading and practical abilities requires active engagement by older adults. However, research indicates that excessive protective behavior or the assumption of family responsibilities may deprive older adults of opportunities to act independently (26, 27), reducing their chances of practicing relevant skills and leading to functional decline. This hypothesis aims to examine the potential negative impact of overprotective behaviors on family care.

*H4*: Orientation to time and place positively influences reading and praxis ability; *H5*: Reading and praxis ability positively influences memory and calculation ability; *H6*: Memory and calculation ability positively influences orientation to time and place. Together, H4, H5, and H6 illustrate a self-reinforcing positive cycle among core cognitive abilities. Specifically, a stronger orientation toward time and place provides the necessary foundation for effectively performing reading and practical tasks, thereby exerting a positive influence on reading and praxis abilities. Engagement in reading and practical activities exercises attention and memory. Improvements in these capacities are essential for processing and integrating complex temporal and spatial information, which subsequently supports and enhances the accuracy and efficiency of temporal and spatial orientations.

*H7*: The level of social isolation negatively influences naming ability. Naming ability is a core component of linguistic function and is strongly influenced by long-term language environment and educational attainment. Lower levels of social isolation imply more frequent language interactions with the external environment, which may theoretically enrich vocabulary input and communication opportunities, thereby facilitating naming abilities.

*H8*: Family support positively influences naming ability. Family is the most frequent setting for language interaction among older adults. Daily conversations with family members can reinforce naming recall through word repetition and contextual association. Emotional exchanges within family support may also indirectly improve lexical retrieval. Hence, we hypothesized that family support would have a positive effect on naming abilities.

*H9–H11*: Lower social isolation would be positively associated with orientation to time and place, reading and praxis, memory, and calculation abilities. *H9*: The level of social isolation negatively influences orientation to time and place; *H10*: The level of social isolation negatively influences reading and praxis ability; *H11*: The level of social isolation negatively influences memory and calculation ability. A lower level of social isolation indicates that older adults are more likely to participate in social and community group activities such as card games and group outings. These activities often involve judgment of time and space and provide more opportunities for practical operations. Long-term participation may directly exercise memory and calculation abilities.

*H12*: Family support positively influences orientation to time and place. Daily reminders and structured daily routines within family support, such as noting the current date, fixed mealtimes, and scheduled walks, can help older adults establish a regular framework for temporal and spatial cognition. Thus, it was hypothesized that family support would positively influence orientation toward time and place.

### 2.2 Study design and population

#### 2.2.1 Design

**Figure 2:**
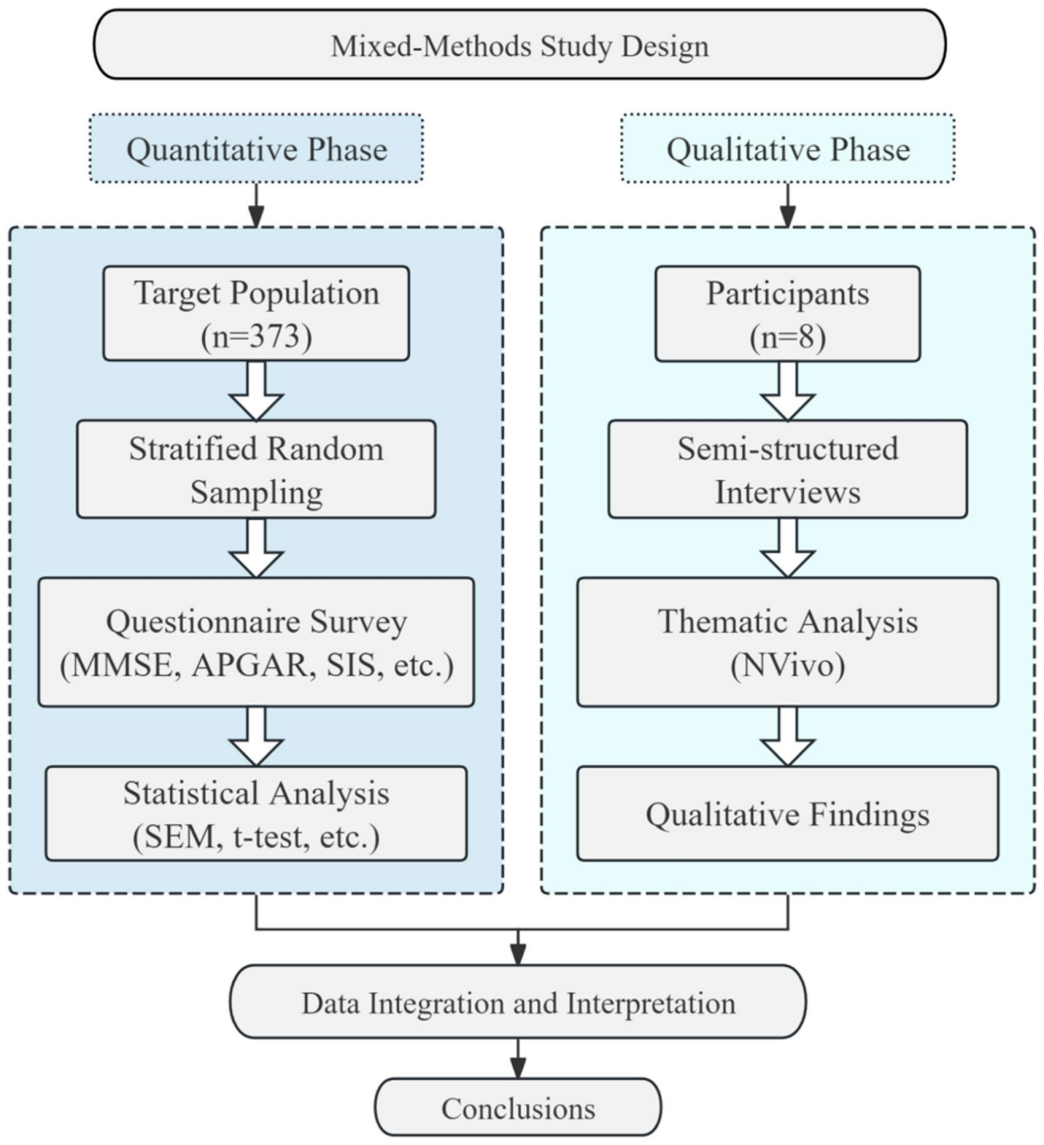
Mixed-methods study design flowchart.

A mixed-methods approach was employed, combining a quantitative cross-sectional survey with a qualitative phenomenological inquiry. The overall design, integrating the quantitative and qualitative phases, is summarized in Figure 2.

We employed a mixed-methods approach, incorporating a quantitative survey on the cognitive levels and family social support among community-dwelling older adults with MCI, along with a qualitative investigation into the key stakeholders involved in providing health management services for this population. Hangzhou, Zhejiang Province, was designated as the research field. As one of the pioneering cities in China to implement community-based pilot programs for MCI interventions and given its advanced demographic aging, Hangzhou offers strong regional representativeness for the present investigation.

The quantitative component assessed the cognitive status of older adults in the communities and examined their current state of family and social support. By analyzing the relationship between family social support and cognitive function, we explored the pathways through which family social support influences cognitive levels, thereby proposing optimization strategies from familial and social perspectives within a multistakeholder collaborative governance framework.

Semi-structured interviews were conducted with community and family physicians to gain insights into different service providers. These interviews aimed to elucidate the current state of health management for older adults with MCI in community settings as well as the challenges, key issues, and practical experiences related to tripartite collaboration among family members, social workers, and physicians.

#### 2.2.2 Study population and sample size

##### 2.2.2.1 Questionnaire survey

A stratified random sampling method was employed to identify residents aged 60 and above from various districts of Hangzhou. Based on a previous study (28), the crude prevalence of MCI among older adults in Zhejiang Province was 15.18%. With a significance level (*α*) set at 0.05 and a margin of error (E) set at 4%, the sample size was calculated using the formula:


n=Zα2∗p∗(1−p)E2

Accounting for a 15% non-response rate, the minimum required sample size was 326. In total, 378 questionnaires were distributed. Based on the proportional distribution of the population aged 60 and above across different districts—as reported in the 2024 Hangzhou Statistical Yearbook—the sample sizes per district were as follows: Shangcheng District (73), Gongshu District (73), Xihu District (48), Qiantang District (18), Binjiang District (15), and Xiaoshan District (103). Ultimately, 373 valid questionnaires were collected, yielding a response rate of 98.68%, which met the sample size requirement.

##### 2.2.2.2 Semi-structured interviews

Based on the research objectives, semi-structured interviews were conducted with incumbent social workers and physicians from community health centers located in the subdistricts where the questionnaire was administered. Before the interviews, the purpose of the study was explained to ensure that the participants had a clear understanding of the research objectives. The audio of the interviews was recorded in entirety and all participants were anonymized. Eight individuals were interviewed, including four social workers and four physicians from the Zhanongkou (Shangcheng District), Chengxiang (Xiaoshan District), and Cangqian (Yuhang District) subdistricts.

### 2.3 Quantitative research

#### 2.3.1 Dependent variable

The dependent variable was cognitive function, assessed using the Mini-Mental State Examination (MMSE), which is widely used to evaluate cognitive impairment because of its brevity, ease of administration, and high participant acceptance. The scale consists of 11 items, including orientation to time and place, registration, attention and calculation, recall, naming, repetition, ability to follow a three-stage command, comprehension, and ability to write a sentence or copy a design. The total score ranges from 0 to 30, with higher scores indicating better cognitive function. According to the Chinese Guidelines for the Diagnosis and Treatment of Dementia and Cognitive Impairment (2020) (28), an MMSE score between 27 and 30 is considered normal, whereas a score below 27 suggests cognitive impairment.

#### 2.3.2 Independent variables

##### 2.3.2.1 Socio-demographic factors

These included general demographic characteristics and risk factors for MCI. The factors covered were sex, age, educational level, marital status, living arrangement, alcohol consumption, smoking, physical exercise, and family history of dementia.

##### 2.3.2.2 Family support

The level of family support was measured from the perspective of family function, reflecting participants’ subjective satisfaction with their families’ function. This study used the Family APGAR (Adaptation, Partnership, Growth, Affection, and Resolve) Index to assess the level of family support among community-dwelling older adults. The APGAR is a self-report instrument designed to evaluate family function and has been extensively applied in research involving diverse populations both internationally and in China (29–31). This instrument assesses five dimensions of family functioning: adaptation, partnership, growth, affection, and resolution. Each dimension is represented by a single item rated on a 3-point scale: “almost always” (2 points), “some of the time” (1 point), and “hardly ever” (0 points). The scores for each dimension were summed to yield a total score ranging from 0 to 10. A total score of 7–10 indicated good family function; 4–6, moderate family dysfunction; and 0–3, severe family dysfunction.

##### 2.3.2.3 Social isolation

In this study, the level of social isolation refers to the degree of connection that older adults maintain with the external world as assessed using the Social Isolation Scale in Older Adults (SIS). This scale integrates Roy’s Adaptation Model and social network theory to measure both the subjective and objective aspects of social isolation in older adults (32). Various versions of the SIS have been widely used in the general population (33, 34). The Chinese version has demonstrated good reliability and validity, proving to be a simple, rapid, and reliable assessment tool. To derive more precise conclusions, this study utilized the objective subscale of the SIS—the “connectedness” dimension—to evaluate the frequency of contact with family, friends, and neighbors. Items were rated on a 5-point Likert scale ranging from 0 to 4, with higher scores indicating stronger connectedness.

#### 2.3.3 Reliability and validity

##### 2.3.3.1 Reliability analysis

A reliability analysis was conducted for continuous variables, including the level of family support, level of social isolation, and MMSE scores. The Cronbach’s alpha coefficient for the scale was 0.724, with values between 0.6 and 0.8 indicating good internal consistency, confirming that the questionnaire used in this study has acceptable reliability (35).

##### 2.3.3.2 Validity analysis

The Kaiser–Meyer–Olkin (KMO) test and Bartlett’s test of sphericity were used to evaluate the validity of the continuous variables, including the level of family support, level of social isolation, and MMSE scores. Bartlett’s test yielded an approximate chi-square value of 2285.911 with a significance level of *p* < 0.001. The KMO value was 0.777. Based on Kaiser’s criteria, the KMO results suggested that the items were suitable for factor analysis.

### 2.4 Qualitative study

#### 2.4.1 Interviews

Based on the research objectives, incumbent social workers and family physicians from community health centers located in the subdistricts where the questionnaire was administered were selected for semi-structured interviews. Before the interviews, the purpose of the study was explained to ensure that the participants understood the research objectives. Each interview lasted 30 min.

#### 2.4.2 Interview time and location

The researchers and interviewees agreed on a suitable time for the interviews. Interviews were conducted face-to-face between February and April 2025. Interviews with social workers were conducted in their respective subdistrict offices, and interviews with physicians were held in the corresponding community health centers.

#### 2.4.3 Interview outline

The interviews were structured around different themes, with separate outlines for social workers and physicians. The specific contents are as follows. The interview outline for social workers consisted of three parts:

1. Existing measures for health management of older adults with MCI in the community include the number of older adults with MCI, how they are identified, and whether the community provides services such as emotional comfort, daily life assistance, outdoor activity support, and safety monitoring.
2. The use of digital tools in daily work, including whether digital tools are used and how they are applied, and the evaluation of these tools regarding usability and comfort.
3. Collaboration with physicians and family members, including communication methods and frequency with physicians, division of responsibilities, and any collaboration issues due to poor communication or unclear duties. It also covers communication methods and frequency with family members, whether services such as psychological support or training are provided to families, and difficulties encountered in collaborating with families.

The interview outline for physicians also included three parts, maintaining a similar structure but adjusted to reflect their responsibilities:

1. The current status of health management for older adults with MCI includes the prevalence of MCI in the community and the provision of services, such as health management, physical care, rehabilitation, and referral.
2. Use of digital tools in health management work, with evaluations of these tools based on data quality, user experience, cost, and effectiveness.
3. Collaboration with social workers and family members: communication methods, frequency with social workers, division of responsibilities, and collaboration challenges. It also covers interactions with families, cooperation in developing and implementing treatment and rehabilitation plans, whether training is provided to families, and difficulties faced in collaborating with families.

### 2.5 Quality control and ethical considerations

#### 2.5.1 Cross-sectional survey

A multistage quality control procedure was implemented to ensure high data quality. (1) Pre-survey training: All investigators received standardized training to ensure the consistent use of language and assessment scales. (2) Pilot survey: A pilot survey was conducted with 30 randomly selected residents of Hangzhou. This helped to identify and revise ambiguous or difficult-to-understand items. The reliability and validity of the scales were tested and improvements were made based on the pilot results. (3) Onsite supervision: During the formal survey, the investigators assisted the participants in completing the questionnaire and provided clarifications when needed to minimize misunderstandings. All questionnaires were checked on site for completeness. Supervisors conducted daily reviews to promptly identify and correct errors. (4) Data Entry and Validation: Invalid questionnaires were excluded after data collection. The responses were examined for logical consistency. Two staff members independently entered the data into electronic databases, and cross-checking was performed to ensure accuracy.

#### 2.5.2 Quality control in qualitative interviews

A semi-structured interview guide was used to maintain focus within the research scope. Before the interviews, the participants were informed of the study purpose to facilitate relevant responses.

The audio recordings were transcribed verbatim and divided into two sets, each assigned to a separate analyst. Initial coding was performed line-by-line with minimal interpretation, relying on the participants’ original wording. The two analysts then compared and discussed their coding results, and iteratively refined the themes until a consensus was reached. To ensure theoretical saturation, open and axial coding were applied to the last participant’s transcript. No new themes emerged, indicating that the sample size was sufficient.

#### 2.5.3 Ethical approval

The study was conducted in accordance with the principles of the Declaration of Helsinki. Ethical approval was obtained from the Ethics Committee of the Hangzhou Normal University (Approval No. 2021–1,147). Written informed consent was obtained from all participants.

### 2.6 Statistical analysis

#### 2.6.1 Descriptive analysis

Categorical variables, including basic demographic characteristics, such as sex, age, and education level, were described using frequencies. According to Kline (36), sample data can be considered normally distributed when skewness is within ±3 and kurtosis is within ±10. In this study, continuous variables such as the Family APGAR Index, level of social isolation, and MMSE scores fell within these ranges. Therefore, these variables are presented as means and standard deviations. All analyses were performed using SPSS Statistics version 26.0 (IBM Corp., Armonk, NY).

#### 2.6.2 Comparative analysis

Differences in MMSE scores across demographic characteristics were examined using appropriate statistical tests. Independent sample t-tests were used for binary variables, such as sex, and one-way analysis of variance (ANOVA) was applied for multi-category variables, such as education level. Analyses were conducted using SPSS Statistics version 26.0 (IBM Corp., Armonk, NY).

#### 2.6.3 Structural equation modeling

Structural equation modeling (SEM) was chosen for its ability to analyze latent variables. The core constructs of family support, social isolation, and cognitive domains were represented as latent variables, measured by their respective multi-item scales. Furthermore, the hypothesized model involved multiple independent and dependent variables with potential mediating effects and feedback loops. Traditional regression methods are inadequate for examining complex relationships between multiple causal paths and latent mediators. Therefore, this study employed AMOS (version 24.0; IBM Corp., Armonk, NY) to investigate the pathways through which the level of social isolation and family support influences specific cognitive domains (assessed via the MMSE: orientation to time and place, memory and calculation, naming ability, and reading and praxis) among older adults with MCI. The analysis focused on the direct effects of social isolation on family support, the direct effects of both on cognitive domains, and the possible interactions among the cognitive domains. This approach helped to quantify the relative importance of each factor and identify the key drivers of cognitive function in this population, thereby providing an empirical basis for optimizing collaborative governance strategies.

In the SEM analysis, we assessed multicollinearity prior to model fitting by examining the variance inflation factor (VIF) for all observed variables, with values below 5 indicating no substantial multicollinearity. During the model specification process, modification indices (MI) were consulted to identify potential areas for model improvement. Only theoretically justifiable modifications with a large MI value (MI > 4) were considered to enhance model fit without compromising theoretical integrity.

#### 2.6.4 Thematic analysis

Qualitative data were analyzed using thematic analysis. This method aims to identify, analyze, and report patterns (themes) within textual data (37). Interview recordings were transcribed verbatim and inductive thematic analysis was conducted using NVivo (version 11; QSR International, Burlington, MA). The process involved three levels of coding to identify key themes related to the current state and challenges of multistakeholder collaboration among social workers, physicians, and family members. The results provide a foundation for further optimization of collaborative governance.

## 3 Results

### 3.1 Current situation analysis

#### 3.1.1 Demographic characteristics

A total of 373 valid questionnaires were collected. Demographic characteristics revealed that females (64.9%) outnumbered males (35.1%), which may be related to the study focus on older adults and women’s longer life expectancies. In terms of age distribution, most participants were aged 70–79 years (40.2%), and those aged 80 and above accounted for 24.6%, indicating a predominance of older age groups. Educational attainment was predominantly primary school (38.6%) and junior high school (30.8%), with only 4.8% holding a bachelor’s degree or higher, which is consistent with the general education background of this population. Regarding marital status, 72.9% were married and 23.6% were widowed. In terms of living arrangements, 38.9% lived with their spouses, 18.2% lived alone, and 22.3% lived only with their children. With respect to health behaviors, 86.3% of the older adults reported engaging in regular physical exercise, while the proportions of smokers (13.7%) and alcohol consumers (20.9%) were relatively low. Only 5.4% of the population had a family history of dementia. The detailed information is presented in Table 1.

**Table 1:** Analysis of demographic characteristics.

Variable	Option	Frequency	Percentage
Gender	Male	131	35.10%
	Female	242	64.90%
Age (years)	<60	11	2.90%
	60–69	120	32.20%
	70–79	150	40.20%
	80–89	77	20.60%
	≥90	15	4.00%
Educational level	Illiterate	32	8.60%
	Primary school	144	38.60%
	Junior high school	115	30.80%
	High school	64	17.20%
	Bachelor’s degree or above	18	4.80%
Marital status	Married	272	72.90%
	Never married	4	1.10%
	Divorced	9	2.40%
	Widowed	88	23.60%
Living arrangement	Living alone	68	18.20%
	Living with spouse	145	38.90%
	Living with children	83	22.30%
	Living with both children and spouse	77	20.60%
Alcohol consumption	Yes	78	20.90%
	No	295	79.10%
Smoking	Yes	51	13.70%
	No	322	86.30%
Regular exercise habit	Yes	322	86.30%
	No	51	13.70%
Family history of dementia	Yes	20	5.40%
	No	353	94.60%

#### 3.1.2 Family APGAR index

The mean score of the Family APGAR Index among the respondents was 8.59 ± 2.12, indicating good family function. The item “I am satisfied with the way my family members and I spend time together” received the highest score (1.81 ± 0.46), while the item “When I want to start new activities or make changes, my family accepts and supports me” received the lowest score (1.62 ± 0.61). The detailed scores for all items are presented in Table 2.

**Table 2:** Scores of the family APGAR index.

Item	Mean	Standard deviation
I am satisfied with the help I receive from my family when I am in difficulty.	1.71	0.58
I am satisfied with the way my family discusses and shares problems with me.	1.72	0.55
When I want to start new activities or make changes, my family accepts and supports me.	1.62	0.61
I am satisfied with the way my family expresses emotions and responds to my feelings.	1.73	0.52
I am satisfied with the way my family members and I spend time together.	1.81	0.46
Total APGAR Score	8.59	2.12

#### 3.1.3 Social isolation

The mean score of the level of social isolation among the respondents was 8.31 ± 2.86. The item “Face-to-face communication or interaction at least once a month” received the highest score (3.25 ± 1.018), while “Confiding in others” received the lowest score (2.38 ± 1.25). “Feeling close to someone on a personal level” scored 2.68 ± 1.158. The detailed results are presented in Table 3.

**Table 3:** Scores of the level of social isolation.

Item	Mean	Standard deviation
Face-to-face communication or interaction at least once a month?	3.25	1.018
Confiding in others?	2.38	1.25
Feeling close to someone on a personal level?	2.68	1.158
Total score	8.31	2.86

#### 3.1.4 MMSE scores

The mean MMSE score of the surveyed population was 24.68 ± 4.6, which falls within the range suggesting possible cognitive impairment. Detailed scores for each item are listed in Table 4.

**Table 4:** MMSE scores.

Item	Minimum	Maximum	Mean	Standard deviation
Orientation to time	1	5	4.52	1.05
Orientation to place	1	5	4.57	0.96
Registration	1	3	2.8	0.52
Attention and calculation	1	5	3.13	1.65
Recall	1	3	2.17	0.88
Naming 1	1	1	0.99	0.09
Naming 2	1	1	0.99	0.09
Repetition	1	1	0.73	0.44
Three-stage command	1	3	2.68	0.69
Reading	1	1	0.84	0.36
Writing	1	1	0.61	0.49
Copying	1	1	0.65	0.48
Total MMSE Score	12	30	24.68	4.6

### 3.2 Health management for older adults with MCI

#### 3.2.1 Univariate analysis

Differences in MMSE scores across various demographic factors were analyzed using independent sample t-tests for binary variables (sex, alcohol consumption, smoking, exercise habit, and family history of dementia) and one-way ANOVA for multi-category variables. The results revealed significant differences in MMSE scores based on sex, age, educational level, marital status, living arrangement, smoking, and exercise habits. In terms of education level, MMSE scores were significantly lower in the “illiterate” and “primary school” groups compared to the “junior high school,” “high school,” and “bachelor’s degree or above” groups. Regarding marital status, the “widowed” group had significantly lower MMSE scores than the “married” group. Significant differences were also observed between older adults with and without exercise habits. The detailed results are presented in Table 5.

**Table 5:** Influence of demographic factors on MMSE scores.

Variable	Group	Mean	Standard deviation	*F*	*p*-value	*Post-hoc* tests (LSD)
Gender	1. Male	25.47	4.08	3.3314	0.014	
	2. Female	24.26	4.81			
Age (years)	1. <60	26.73	1.62	6.856	<0.001	1 > 4, 1 > 5, 2 > 4, 3 > 4
	2. 60–69	25.63	3.76			
	3. 70–79	25	4.65			
	4. 80–89	22.82	4.99			
	5. ≥90	22	5.93			
Educational level	1. Illiterate	18.78	4.72	28.242	<0.001	1 < 2,3,4,5; 2 < 3,4,5
	2. Primary school	23.65	4.35			
	3. Junior high school	26.37	3.51			
	4. High school	26.48	3.93			
	5. Bachelor’s or above	26.28	3.8			
Marital status	1. Married	25.31	4.36	6.914	<0.001	1 > 4
	2. Never married	24.5	3.32			
	3. Divorced	24.22	3.73			
	4. Widowed	22.81	4.97			
Living arrangement	1. Living alone	23.57	4.91	4.814	0.003	2 > 1,3; 4 > 1,3
	2. Living with spouse	25.06	4.57			
	3. Living with children	23.77	4.49			
	4. Living with both	25.95	4.12			
Smoking	1. Yes	25.76	3.65	5.466	0.032	
	2. No	24.51	4.71			
Regular exercise habit	1. Yes	24.98	4.48	2.083	0.001	
	2. No	22.78	4.93			

#### 3.2.2 Structural equation modeling

##### 3.2.2.1 Exploratory factor analysis

Given the number of included explanatory variables, factor analysis was employed for data reduction. As indicated by the validity test results (KMO = 0.777), the data were suitable for factor analysis. As the items of the MMSE scale have different maximum possible scores, Z-score standardization was applied prior to factor analysis to transform the data into a standard normal distribution, with a mean of 0 and a standard deviation of 1. A principal component analysis was used to extract factors. After performing a varimax orthogonal rotation, six components with eigenvalues greater than one were retained. The cumulative variance explained by these components reached 63.42%, which exceeded the 60% threshold, indicating that the extracted factors sufficiently captured the information in the data. The eigenvalues and variance contribution rates for each component are listed in Supplementary Table 1.

The extracted factors were named as follows. Factor 1 was named family support, comprising items from the Family APGAR Index (Adaptation, Partnership, Growth, Affection, and Resolve); Factor 2, social isolation, consisting of items from the Social Isolation Scale (contact with family, friends, and neighbors); Factor 3, orientation to time and place, including the two MMSE items assessing orientation to time and place; Factor 4, memory and calculation, encompassing the items for registration, attention and calculation, and recall; Factor 5, naming ability, containing two items evaluating naming; and Factor 6, reading and praxis, including items for repetition, three-stage command, reading, writing, and copying. The rotated component matrixes are listed in Supplementary Table 2.

##### 3.2.2.2 Confirmatory factor analysis

**Figure 3:**
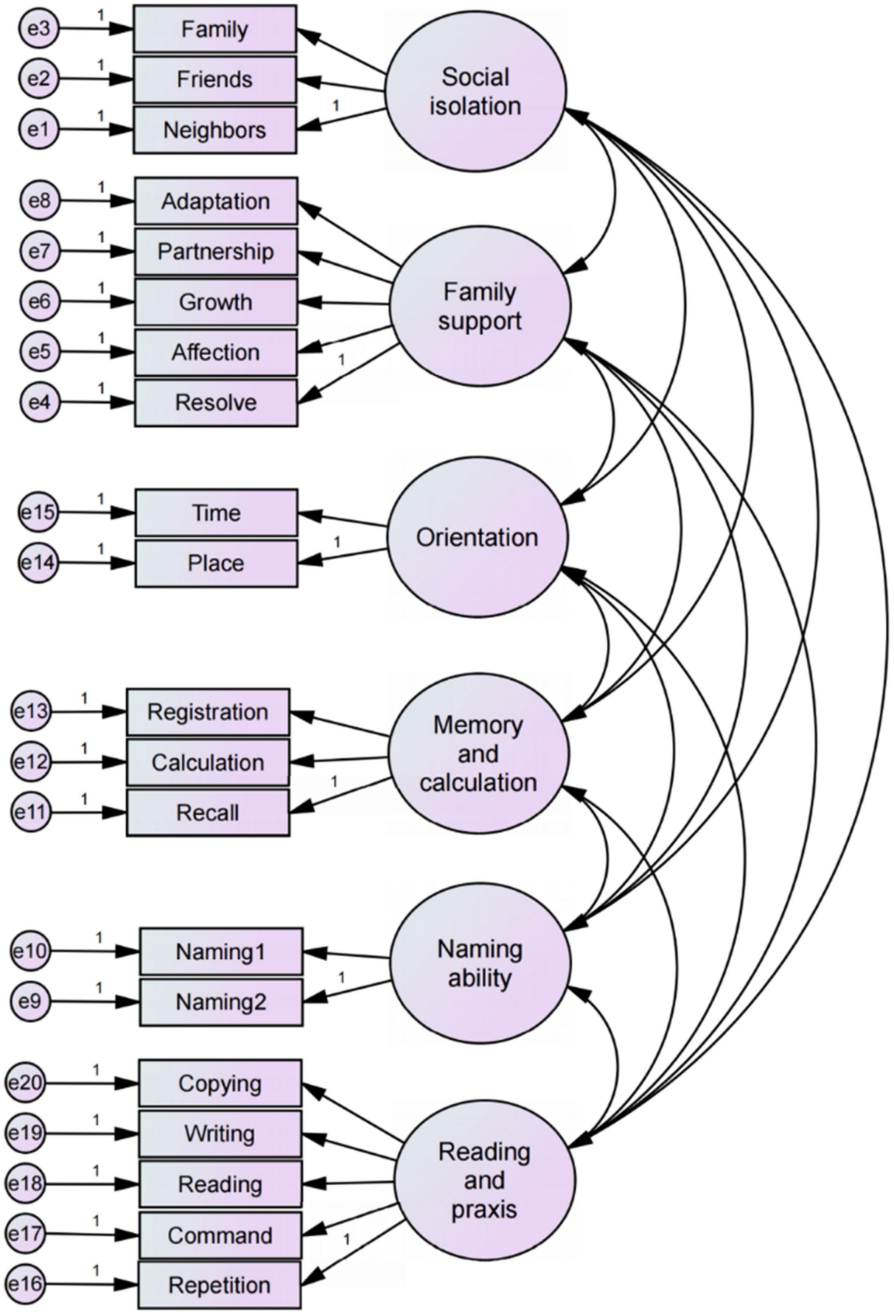
Confirmatory factor analysis (CFA) model.

Following exploratory factor analysis, which identified the initial factor structure and its corresponding measurement items, confirmatory factor analysis (CFA) was conducted to further validate the fit between the measurement items and the hypothesized model. The CFA model is shown in Figure 3.

As shown in Supplementary Table 3, the model fit indices indicate that the chi-square/degrees of freedom ratio (CMIN/DF) was 2.197, which fell within the excellent range of 1–3. The root mean square error of the approximation (RMSEA) was 0.057, indicating a good fit. All other indices met the recommended standards. Therefore, the MMSE-Family Social Support CFA model demonstrated a good overall fit.

The convergent validity and composite reliability of each dimension were further examined under the precondition of adequate model fit. Factor loadings greater than 0.4 are considered acceptable, and all measurement items in this study met this criterion. The Average Variance Extracted (AVE) and Composite Reliability (CR) were calculated for each dimension. An AVE value greater than 0.36 is considered acceptable, while a value exceeding 0.5 is ideal (38). The AVE values for the five latent variables in this study met the standard, although the AVE for the memory and calculation dimensions were slightly below 0.36. However, an AVE value marginally below the threshold remains acceptable if CR exceeds 0.6 (39). Furthermore, when a latent variable was measured by fewer than six items, a CR value greater than 0.6 indicates acceptable reliability (40). The CR values for the four latent variables met this criterion. The CR values for Naming Ability and Orientation to time and place fell between 0.5 and 0.6, which may be attributed to the limited number of measurement items for these constructs. Nevertheless, these dimensions were retained in the analysis because of their foundation in the authoritative MMSE, whose item structure aligns with established medical standards and maintains analytical rigor. The detailed results are presented in Table 6.

**Table 6:** Tests of convergent validity and composite reliability for each dimension (*n* = 373).

Path	Estimate	AVE	CR
Contact with neighbors ← Social isolation	0.788	0.555	0.785
Contact with friends ← Social isolation	0.844		
Contact with family ← Social isolation	0.575		
Resolve ← Family support	0.793	0.533	0.849
Affection ← Family support	0.774		
Growth ← Family support	0.578		
Partnership ← Family support	0.805		
Adaptation ← Family support	0.676		
Naming 2 ← Naming ability	0.761	0.382	0.535
Naming 1 ← Naming ability	0.431		
Recall ← Memory and calculation	0.488	0.355	0.616
Attention and calculation ← Memory and calculation	0.718		
Registration ← Memory and calculation	0.557		
Orientation to place ← Orientation to time and place	0.77	0.436	0.599
Orientation to time ← Orientation to time and place	0.528		
Repetition ← Reading and praxis	0.481	0.427	0.785
Three-stage command ← Reading and praxis	0.744		
Reading ← Reading and praxis	0.742		
Writing ← Reading and praxis	0.668		
Copying ← Reading and praxis	0.595		

Based on the analysis results presented in Table 7, it can be observed that in this discriminant validity test, the standardized correlation coefficients for all dimensions, except for the memory and calculation dimensions, were lower than the square root of the corresponding AVE value for that dimension. Although one factor fell slightly below the standard, most factors met the criterion and remained within the acceptable range (41). This indicated that the various dimensions exhibited good discriminant validity.

**Table 7:** Discriminant validity test (*n* = 373).

Dimension	Social isolation	Family support	Naming ability	Memory and calculation	Orientation to time and place	Reading and praxis
Social isolation	**0.555**					
Family support	0.406	**0.533**				
Naming ability	0.039	−0.01	**0.382**			
Memory and calculation	0.013	0.059	−0.039	**0.355**		
Orientation to time and place	0.025	−0.049	0.007	0.533	**0.436**	
Reading and praxis	0.017	−0.146	0.099	0.654	0.502	**0.427**
√AVE	0.745	0.73	0.618	0.596	0.66	0.653

The bold values are the AVE values corresponding to each dimension.

##### 3.2.2.3 Correlation analysis

Correlation analysis was conducted on the scores for family support, social isolation, orientation to time and place, memory and calculation, naming ability, reading, and praxis. Significant correlations were found between orientation to time and place and memory and calculation and reading and praxis at a significance level of 0.05. There was a significant correlation between family support and social isolation. At a significance level of 0.1, a correlation was found among family support, reading, and praxis. The detailed results are presented in Table 8.

**Table 8:** Correlation analysis among dimensions (*n* = 373).

Dimension	Family support	Social isolation	Orientation to time and place	Memory and calculation	Naming ability	Reading and praxis
Family support	1					
Social isolation	0.213** (*p* < 0.001)	1				
Orientation to time and place	−0.024 (*p* = 0.65)	−0.03 (*p* = 0.56)	1			
Memory and calculation	0.116* (*p* = 0.025)	0.054 (*p* = 0.296)	0.202** (*p* < 0.001)	1		
Naming ability	0.024 (*p* = 0.648)	0.045 (*p* = 0.384)	0.029 (*p* = 0.58)	−0.001 (*p* = 0.983)	1	
Reading and praxis	−0.098 (*p* = 0.058)	0.044 (*p* = 0.4)	0.338** (*p* < 0.001)	0.301** (*p* < 0.001)	0.034 (*p* = 0.516)	1

**represents significance at the 0.01 level (two-tailed), and *represents significance at the 0.05 level (two-tailed).

##### 3.2.2.4 Structural equation modeling (SEM)

**Figure 4:**
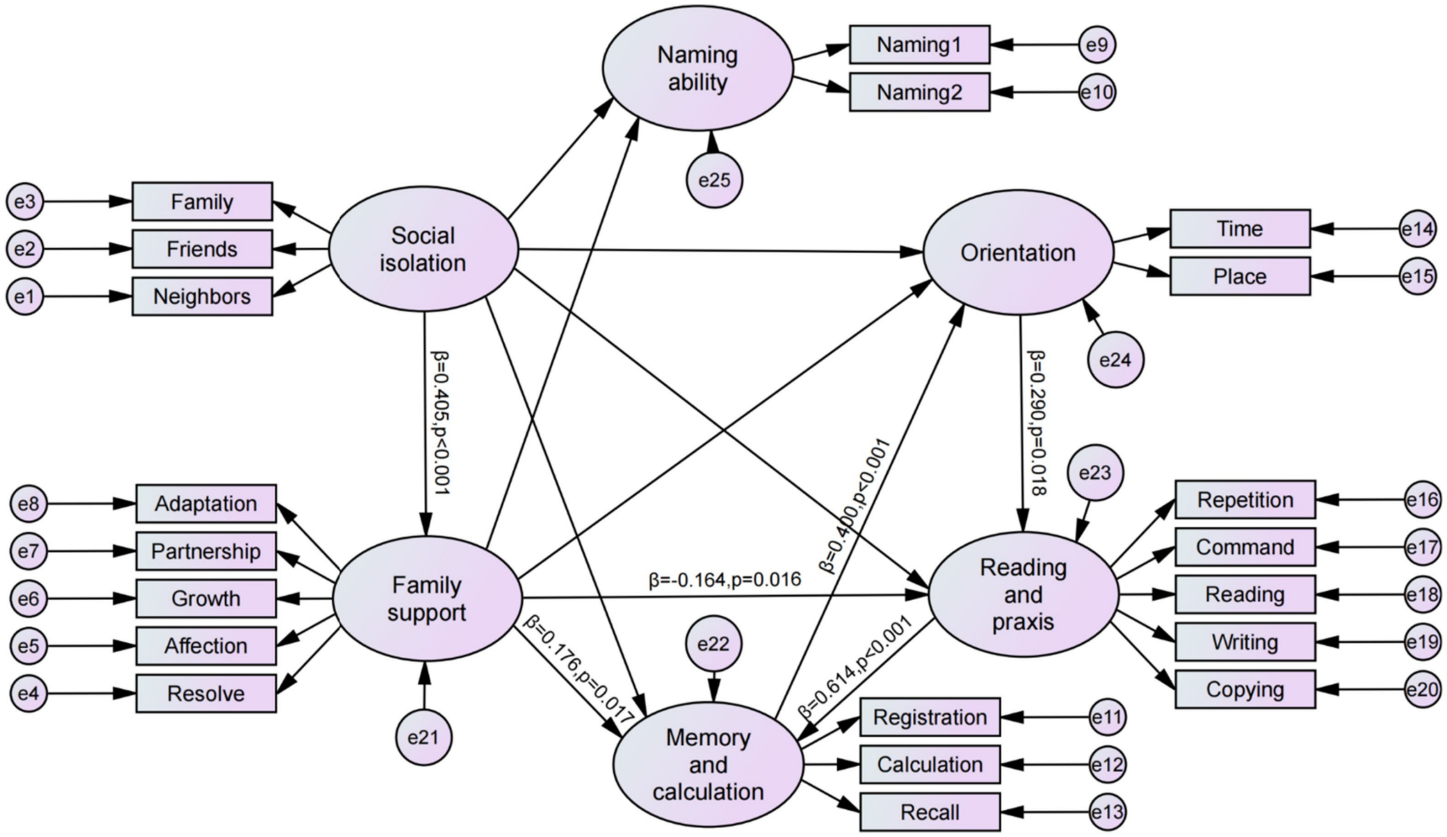
Structural equation model (SEM).

Based on the factor analysis, the constructs of family support, social isolation, orientation to time and place, memory and calculation, naming ability, and reading and praxis were incorporated into the SEM. The model paths were partially adjusted according to the correlation analysis results. The final SEM image is shown in Figure 4.

Common model fit indices include the chi-square/degrees of freedom ratio (CMIN/DF), RMSEA, incremental fit index, Tucker–Lewis index (TLI), and comparative fit index. For this model, the CMIN/DF was 2.164, and the RMSEA was 0.056, both of which met the recommended standards. All other indices satisfied the required criteria. The model results indicated that a lower level of social isolation (with higher scores indicating a lower risk of isolation) positively influenced family support (path coefficient = 0.405, *p* < 0.001). Family support positively influenced memory and calculation ability (path coefficient = 0.176, *p* < 0.05) and negatively influenced reading and praxis ability (path coefficient = −0.164, *p* < 0.05). Orientation to time and place positively influenced reading and praxis abilities (path coefficient = 0.29; *p* < 0.05). Reading and praxis abilities positively influenced memory and calculation abilities (path coefficient = 0.614, *p* < 0.001), while memory and calculation abilities positively influenced orientation to time and place (path coefficient = 0.4, p < 0.001). The results of the structural equation model are summarized in Table 9, which presents the significant pathways. A comprehensive listing of all standardized path coefficients, standard errors, confidence intervals, and exact *p*-values for every hypothesized path (H1-H12) is provided in Supplementary Table 4.

**Table 9:** Significant path relationships in the structural equation model (*n* = 373).

Path	Estimate	S.E.	C.R.	*P*
Family support ← Social isolation	0.405	0.025	6.423	<0.001
Memory and calculation ← Family support	0.176	0.098	2.396	0.017
Reading and praxis ← Family support	−0.164	0.089	−2.417	0.016
Reading and praxis ← Orientation to time and place	0.29	0.077	2.363	0.018
Memory and calculation ← Reading and praxis	0.614	0.12	5.152	<0.001
Orientation to time and place ← Memory and calculation	0.4	0.192	3.304	<0.001

##### 3.2.2.5 Hypothesis testing results of the SEM

Based on the model fit evaluation and path analysis, it is assumed that H1–H6 are supported, while H7–H12 are not supported, and the results of the hypothesis testing are summarized in Supplementary Table 5.

### 3.3 Interview results

#### 3.3.1 Basic information about the interviewees

This study selected subdistricts in which questionnaires were distributed based on the research objectives, and conducted semi-structured interviews with family physicians from community health service centers and social workers. Of the eight participants, social workers were assigned codes A1–A4 and family physicians were assigned codes B1–B4. The respondents’ basic demographic information is presented in Supplementary Tables 6, 7. Approximately 87 min of audio recording yielded over 20,000 words of transcribed textual data.

#### 3.3.2 Three-level coding of social worker interviews

We employed thematic analysis, a method aimed at identifying, analyzing, and reporting themes within qualitative data. The analysis followed a structured six-step process: familiarization with the data, generating initial codes, searching for themes, identifying themes, naming themes, and producing reports. The analysis was applied to the interview data collected from the social workers, resulting in the extraction of 37 open codes, 17 axial codes, and four selective codes. Supplementary Table 8 lists the initial codes and representative original statements. Owing to space limitations, only one illustrative quotation is provided for each open code.

The obtained initial codes were verified and consolidated, and primary code units with similar connotations were categorized to establish secondary codes, which were further synthesized and refined to derive the tertiary codes. Table 10 presents the final three-tier thematic coding structure derived from the social worker interviews.

**Table 10:** Final three-tier thematic coding structure from social worker interviews (*n* = 4).

Selective codes	Axial codes	Open codes
Status of health management	Rehabilitation services	Operation by a third-party center, limitations to home-based rehab, Perceived lack of benefit in rehab for the older adult(s), Brain games
	Emotional support	Holiday home visits, organization of recreational and cultural activities, Emotional support
	Daily living support	In-home daily living support services by third parties, meal delivery service
	Safety monitoring services	Manual safety monitoring, long-term care insurance (LTCI)
Digital health management	Negative feedback	Low acceptance, limited interregional data sharing
	Positive feedback	Ease of use, health education promotion, improved situational awareness of elders
	Status of digital technology adoption	Promotion via official social media accounts, health-tracking wristband, smart doorbell
	Barriers to adoption	Low technological adoption among elders
Collaboration with family caregivers	Identified challenges	Lack of family cooperation
	Role delineation	Family-provided daily care
	Routine communication	Home visits, communication via WeChat
	Communication frequency	Weekly, quarterly
	Services for family caregivers	Lack of specialized training, psychological counseling, public educational lectures
Collaboration with family physicians	Identified challenges	Limited direct communication
	Role delineation	Joint home visits, synchronization of patient status updates, health lectures
	Routine communication	Face-to-face communication, communication via WeChat
	Communication frequency	Infrequent communication, contact frequency was situation-dependent

#### 3.3.3 Three-level coding of family physician interviews

The same analytical procedure was applied to interview data collected from family physicians, yielding 37 open codes, 12 axial codes, and four selective codes. Supplementary Table 9 displays the initial-level codes and representative verbatim quotations from physician interviews. Owing to space constraints, an illustrative excerpt was provided for each open code.

The obtained initial codes were verified and consolidated, and primary code units with similar connotations were categorized to establish secondary codes. These secondary codes were then further synthesized and refined to derive the tertiary codes. Table 11 presents the final three-tier thematic coding structure derived from the family physicians’ interviews.

**Table 11:** Final three-tier thematic coding structure from family physicians interviews (*n* = 4).

Selective codes	Axial codes	Open codes
Status of health management	Care	Medically-integrated home care, assessment by community health centers; civil affairs services, LTCI provision by third-party entities
	Health check-ups	Low community participation, lack of equipment, lack of targeted health screenings, implementation of cognitive screening
Digital health management	Negative feedback	Suboptimal accuracy
	Positive feedback	Ease of use, high acceptance, data security, ease of maintenance, workflow enhancement
	Status of digital technology adoption	Wearable devices, electronic health records (EHR)
	Barriers of digital technology adoption	Inability for independent use
Collaboration with family caregivers	Identified challenges	Lack of family cooperation, unrealistic family expectations, time-consuming patient education
	Routine communication	Clinician-family communication at point-of-care、enterprise WeChat
	Services for family caregivers	Shared decision-making for treatment plans, caregiver training, push notifications for health information, push notifications for health information, lack of specialized caregiver training, psychological counseling, teleconsultation
Collaboration with social workers	Identified challenges	Not yet encountered
	Role delineation	Synchronization of patient status updates, facilitating hospital transfers, home visits to special populations, health check-ups、 organizing health lectures
	Routine communication	Context-dependent communication frequency, face-to-face communication, communication via WeChat and phone calls

## 4 Discussion

This study used a mixed-methods design to explore the pathways of collaborative health governance and identify optimization strategies for community-dwelling older adults with MCI in Hangzhou, China. Our findings revealed the dual effect of family support on cognitive function, identified positive feedback loops among cognitive domains, and uncovered key barriers in multi-stakeholder collaboration. The following sections interpret these findings in the context of existing literature, discuss the study’s implications and limitations, and propose directions for future research.

### 4.1 Differences in MMSE performance

This study revealed significant disparities in MMSE performance based on sex, age, educational attainment, marital status, living arrangements, smoking status, and exercise habits. Male participants demonstrated significantly higher MMSE scores compared to female participants, a finding consistent with previous reports (42, 43). This may be attributable to the healthy survival effect, given that men generally exhibit lower survival rates in older populations, a phenomenon that was also reflected in our sample, in which 64.9% were women. These gender disparities may stem from historical inequalities in educational accessibility.

From an age-related perspective, advanced age was significantly associated with lower MMSE scores. Participants in the oldest-old group (aged 80 years and older) consistently scored lower than those in the younger-old group (under 80 years), which aligns with the findings of a previous study (44). Educational level exerted a substantial protective effect on cognitive performance: the illiterate group demonstrated the lowest scores, whereas individuals with a junior high school education or above showed significantly higher MMSE results. These outcomes support the cognitive reserve hypothesis (45), which suggests that higher educational attainment enhances neural plasticity through mechanisms such as increased synaptic density and efficient neural networking, thereby mitigating cognitive decline.

Married individuals demonstrated significantly higher MMSE scores compared to widowed participants. This disparity may stem from cognitive engagement and emotional support afforded by marriage, which are conducive to maintaining cognitive activity. Living arrangements further substantiate this mechanism: older adults living alone showed the lowest scores, whereas those co-residing with a spouse—whether living solely with a spouse or together with both spouse and children—exhibited significantly better performance than those not living with a spouse (e.g., living alone or only with children). Together, these dimensions underscore the role of familial interactions and social isolation in shaping cognitive function, with the presence of a spouse emerging as a particularly salient protective factor. Although both children and spouses constitute family support, the influence of spouses appears to be more pronounced among older adults, which is consistent with previous findings (46).

Overall, these findings indicate that cognitive aging is a multidimensional phenomenon shaped by the interaction of biological, psychological, and social factors. The study further supports the research direction that cognitive health in later life is not merely the outcome of individual pathological processes, but rather a comprehensive reflection of structural conditions accumulated across the life course—such as educational opportunities, gender equality, and social connectedness (47). Therefore, addressing this issue requires the establishment of integrated policy frameworks and community-based intervention systems, positioning education-, gender-, and participation-oriented aging support policies as core strategies for promoting cognitive health among older adults.

### 4.2 Pathways of health management impact

This study employed SEM to examine the complex relationships between family support, social isolation, and cognitive function. Based on these quantitative findings, we elucidated the underlying mechanisms influencing cognitive performance. Lower levels of social isolation (as reflected by higher scores) were significantly associated with higher levels of family support (path coefficient = 0.405, *p* < 0.001). The strength of this association falls within the medium-high range compared with similar studies examining social support and family functioning (48, 49). This pattern may be attributed to the unique characteristics of older adults with MCI: when basic social support—such as monthly face-to-face interaction—is accessible through community and neighborhood engagement, families are relieved from serving as the sole source of emotional companionship. Consequently, they can redirect efforts toward higher-quality support, such as spending meaningful time together. This finding aligns with the compensatory effect in social support theory. Specifically, ample external support reduces the family’s burden as the primary caregiver, freeing up capacity to deliver more consistent and higher-quality care. Consequently, family support can evolve. It shifts from merely providing survival-oriented tasks (e.g., meal delivery) to facilitating development-oriented activities (e.g., encouraging engagement in new experiences). However, within the relational dimension of social isolation, the item “sharing inner feelings” received a relatively low score, suggesting that deep emotional communication remains largely dependent on family caregivers. This finding highlights the need for communities to develop targeted emotional support initiatives, including structured opportunities for in-depth emotional exchanges that can strengthen emotional connectedness, complement familial support roles, and enhance the overall effectiveness of psychosocial care.

SEM indicated that family support exerted a positive effect on memory and calculation abilities (path coefficient = 0.176, *p* < 0.05), yet a negative influence on reading and praxis performance (path coefficient = −0.164, *p* < 0.05). This paradoxical finding offers important insights into the dynamics of family care models. The beneficial effects of family support on memory and calculation are aligned with the frequent cognitive stimulation inherent in family interactions. This observation is consistent with cognitive enrichment theory (24), which posits that ongoing intellectual engagement helps preserve cognitive function by promoting neural plasticity and strengthening synaptic connectivity. Within the familial context, often the most consistent source of social engagement for older adults, shared activities such as recalling past events, managing household budgets, or planning family activities serve as daily exercises for episodic memory, working memory, and arithmetic skills. Thus, these interactions function as protective factors against a decline in related cognitive domains. Conversely, the inhibitory effect on reading and practical skills appears to stem from what may be termed protective substitution: well-intentioned family caregivers, concerned about potential errors or frustration, often take over tasks such as reading instructions or writing notes on behalf of the older adult. This reduces opportunities for self-initiated practice, as reflected in the relatively low scores on MMSE reading and writing tasks. These quantitative findings were corroborated by the interview responses, indicating that family caregivers often prioritize experiential assistance over encouraging autonomous cognitive efforts. These results underscore the need for families to strike a balance between providing emotional and practical support and fostering independence. Encouraging self-directed engagement in daily tasks may help mitigate overprotective tendencies and support holistic cognitive maintenance in older adults with MCI.

We identified a positive feedback loop among three cognitive domains. Orientation directly facilitated reading and praxis (path coefficient = 0.29, *p* < 0.05). In turn, reading and praxis indirectly strengthened orientation by enhancing memory and calculation (indirect effect: 0.614 × 0.4 = 0.246, *p* < 0.001). This closed-loop mechanism offers precise targets for cognitive intervention. The ability to orient to time and place serves as a foundation for executing complex commands involving reading and manipulation (e.g., multi-step instructions or writing tasks). Conversely, the acts of reading and manipulating information—such as during mental calculation—themselves serve to strengthen memory and temporal–spatial orientation. This mechanism explains why older adults with higher scores on the MMSE “orientation to time and place” items also performed better in “reading and praxis” and “memory and calculation” domains. While previous studies have largely focused on identifying the factors influencing global MMSE scores and the psychometric properties of its subscales, the interrelationships among the cognitive domains measured by the MMSE have often been overlooked. The recursive relationships identified in this study provide a theoretical foundation to design multidomain cognitive training protocols. For example, community-based programs could introduce integrated activities such as map-reading exercises that simultaneously engage orientation, reading comprehension, and executive functions to holistically maintain and enhance cognitive health in older adults.

The model did not identify the significant effects of social isolation or family support on naming ability (H7 and H8 were not supported), suggesting that naming ability, a core dimension of linguistic function, may exhibit functional specificity. Li (50) and Turcotte (51) propose that the preservation of language abilities in cognitive function relies heavily on long-term linguistic environmental exposure and cognitive reserves accumulated through educational experiences. Consequently, naming ability appears to be relatively insensitive to short-term fluctuations in social support networks. The relatively low educational attainment of the current sample may partly account for this finding. Furthermore, no significant direct effects of social isolation were observed on orientation, reading, praxis, memory, and calculation abilities (H9–H11), nor was there a significant association between family support and orientation (H12). These findings can be interpreted from several perspectives. First, the core neuropathology of MCI primarily involves regions such as the hippocampus, entorhinal cortex, and posterior parietal cortex (52). Early declines in orientation, complex task performance, and episodic memory are largely driven by these anatomical changes, which may reduce direct sensitivity to broader psychosocial environmental factors such as social isolation or family support in the early stages of cognitive impairment. Second, the MMSE demonstrated lower discriminative accuracy than the Montreal Cognitive Assessment in differentiating between adults with MCI and those without cognitive deficits (53). Moreover, MMSE subscales show inconsistent and occasional inverse associations with MCI progression (54), indicating limited and heterogeneous predictive validity across cognitive domains. These psychometric limitations may compromise the utility of the MMSE in reliably identifying individuals at a high risk of MCI.

This study argues that health governance for the older adult(s) is a multi-level process of community ecological management, in which cognitive health should be situated within the interactive system of individual health capacity, socio-ecological environment, and family caregiving dynamics. Family support and social isolation should not be viewed as static and independent variables, but rather as context-dependent dynamic processes that can both facilitate functional maintenance through cognitive stimulation and hinder autonomous participation through overprotection (55). Training family caregivers to adopt tiered support strategies and encouraging self-engagement among older adults can help balance emotional care with cognitive autonomy. Furthermore, building community-based emotional support networks can compensate for the limitations of intra-family communication and reduce caregiving burdens. Relying on the community health service system, the design of multidomain integrated cognitive training programs can leverage positive feedback mechanisms across cognitive domains to enhance and transfer cognitive functioning among the older adult(s) population.

### 4.3 Collaborative health management

Social workers and community family physicians demonstrated a complementary division of labor and established a preliminary collaborative framework. Social workers primarily provide nonmedical support, including emotional comfort and daily living assistance, while family physicians focus on clinical interventions, such as brain health screenings (B4) and in-home blood collection (B1), as part of their basic medical services. Collaboration between the two groups occurred primarily during joint home visits or health lectures, with daily communication largely initiated in response to changes in a patient’s health status. This pattern reflects ambiguities in role definition and accountability, consistent with the qualitative results from the study by Bian (17).

At the institutional level, community health centers are evaluated based on medical performance indicators such as screening and referral rates, whereas social workers are assessed according to service coverage metrics. The absence of integrated evaluation criteria encompassing both domains has contributed to fragmented care delivery. Technologically, barriers to information sharing persist; electronic health records remain confined to clinical data and do not incorporate updates from social workers on home care dynamics. Additionally, insufficient cross-regional data coordination, particularly when older adults relocate to live with children in different jurisdictions, further undermines the continuity of coordinated interventions. Therefore, an integrated collaborative platform should be established to mitigate existing service discontinuities. This requires reconciling the current divide between clinical metrics and service-oriented indicators by optimizing the joint evaluation system; for instance, by incorporating coordinated intervention coverage into the performance assessments of community health centers. Furthermore, existing electronic health records could be enhanced through the addition of a dynamic home care module, enabling social workers to document non-clinical observations such as dietary patterns and emotional fluctuations. The development of interoperable data interfaces across regions would facilitate the automatic transfer of health information when older adults relocate, thereby addressing critical gaps in data continuity.

Family caregivers constitute the core of daily care—72.9% of married older adults co-reside with their spouses—yet, insufficient caregiving capacity and perceptual biases among family caregivers have made them a central challenge to collaborative governance. Only one physician mentioned “caregiver skill training,” while most social workers indicated a “lack of specialized training programs.” Consequently, families often rely on experiential rather than evidence-based interventions. This explains why despite adequate functional provision, family support demonstrates limited efficacy in promoting positive cognitive outcomes. Furthermore, low patient engagement is a major external barrier for general practitioners to screen and manage MCI (56). Given the characteristics thereof, patient participation is strongly influenced by family willingness. The interview results revealed a polarization in family attitudes toward support services. Some families exhibit resistance to community-based interventions, with a “lack of family cooperation” frequently cited as a major challenge. Others had unrealistically high expectations, requiring family physicians to devote a substantial amount of time for clarification and education. These findings underscore the need for structured caregiver training programs that cover cognitive rehabilitation techniques, emotional regulation, and assistive device use. Attention to the psychological wellbeing of family caregivers is critical. Strengthening collaboration between family caregivers and social workers not only improves care delivery but also benefits both parties’ mental health (57). Therefore, it is essential to facilitate ongoing communication between families and social workers, offering counseling sessions for those experiencing high levels of care-related stress. The establishment of family support groups may help create a supportive community environment through shared experiences and reduce illness-related stigma.

Digital tools have increased the efficiency of health monitoring, and both social workers and doctors have reported that these tools are helpful in their work. For example, they allow a better understanding of older adult conditions and extend the reach of health knowledge dissemination. However, because of the challenges faced by seniors with cognitive impairment, using digital devices is more difficult, and the acceptance of smart devices remains low among this group. This indicates the need to adapt digital tools from general-use designs to more cognition-friendly alternatives. Therefore, collaboration with technology companies is recommended to develop easy-to-use smart devices that incorporate voice-based interactions and passive cognitive monitoring through dialogue analyses. For older adults with trouble using devices independently, a family assisted approach should be adopted. It is also important to consider those who cannot use digital tools by maintaining non-digital options, such as having volunteers make home visits to record health information.

The above findings reveal the systemic complexity and interdependence of collaborative health governance for older adults in the community, which is particularly pronounced among individuals experiencing cognitive decline. At a deeper level, the effectiveness of governance depends not only on the coexistence of medical and social services but also on the degree of structural integration, information sharing, and relational coordination among family physicians, social workers, and family caregivers within a fragmented institutional ecosystem. Consequently, the medical, social, familial, and technological subsystems must transition from parallel operations toward interdependence and coordinated co-evolution.

### 4.4 Practical implications

Based on our findings, we propose the following practical implications for enhancing community-based MCI care:

First, for community managers and policymakers, it is crucial to establish a standardized tripartite collaboration mechanism. This involves creating formal communication channels and shared responsibilities among social workers, family physicians, and families. Joint evaluation metrics that encompass both clinical outcomes (e.g., MMSE scores) and service coverage (e.g., home visit frequency) should be developed to incentivize integrated care.

Second, family caregiver training programs should be institutionalized. Our findings on the dual effects of family support underscore the need to move beyond experiential care. Structured curricula should be developed and delivered by community health centers, covering topics such as cognitive rehabilitation techniques, communication strategies to avoid overprotection, and the use of assistive digital tools. Establishing family support groups can also provide emotional respite and reduce illness-related stigma.

Third, a multi-faceted digital health strategy, informed by empirical evidence, is crucial for equitable and effective MCI management. Recent empirical studies have demonstrated that digital technologies exert a significant positive effect on cognitive functioning in older adults. The reliance on traditional tools can be alleviated by integrating validated digital screeners. A large-scale implementation study in China demonstrated that a voice-recognition-based Digital Cognitive Screener (DCS) achieved high sensitivity (93.1%) and specificity (85.7%) for detecting MCI, showcasing its feasibility and effectiveness in community settings (58). This evidence strongly supports the development of voice-first interfaces as a practical and accurate means to lower the barrier for cognitive assessment and daily interaction. Training in digital health literacy contributes to enhancing digital adaptability among older adults. A quasi-experimental study demonstrated that a digital literacy program significantly improved participants’ cognitive function scores by 1.305 points (*β* = 1.305, *p* = 0.05) compared to the control group (59). This finding confirms that structured community-based training can directly enhance cognitive engagement and effectiveness in using digital tools.

### 4.5 Limitations

This study has several limitations that should be considered when interpreting the results.

First, the cross-sectional design precludes causal inference regarding the relationships between social isolation, family support, and cognitive function. The positive feedback loops we identified, while theoretically sound, require validation through longitudinal studies.

Second, the generalizability of our findings may be limited by selection bias. Our sample was drawn exclusively from Hangzhou, a major metropolitan city in China with relatively advanced aging services and pilot programs for MCI. Consequently, the identified collaboration mechanisms and challenges may not fully represent the situation in smaller cities or rural areas of China, where healthcare resources and community support structures can differ significantly.

Third, the potential influence of the specific cultural context should be considered. The study was conducted within the framework of Chinese family culture and community governance models. The observed dynamics of family support, the role of social workers, and the interaction between families and the healthcare system may be shaped by these contextual factors. Therefore, the direct applicability of our findings to other cultural settings with different family structures, social norms, and welfare systems may be limited.

Forth, the reliance on self-reported scales (e.g., Family APGAR, SIS) may introduce recall bias or social desirability bias. Future research could benefit from incorporating objective measures of social interaction and family function.

Finally, a significant limitation is the absence of the family caregiver’s perspective in our qualitative data. While we interviewed social workers and physicians, the viewpoints of those providing daily care were not captured. This limits our understanding of the core dynamics within the collaborative governance framework. Future studies should prioritize including family caregivers to obtain a holistic view of the challenges and facilitators of multi-stakeholder collaboration.

Despite these limitations, our study possesses notable strengths, including the application of a mixed-methods design that provided both breadth and depth of insight, the use of robust statistical techniques like SEM to test complex pathways, and the timely focus on optimizing community-based governance for a pressing public health issue.

## 5 Conclusion

This study successfully addressed its primary objective by constructing and empirically testing a collaborative governance framework for community-based MCI management. By integrating quantitative findings on the pathways linking social isolation, family support, and specific cognitive domains with qualitative insights into stakeholder collaboration, we identified both key barriers and optimization levers.

Our findings directly inform the proposed tripartite “Social Worker–Physician–Family” model. The quantitative results underscore the need for this collaboration: family support must be optimized to harness its positive effects on memory while mitigating its negative impact on practical skills, and community efforts should focus on reducing social isolation to strengthen the overall support system. The identified positive feedback loops among cognitive domains further justify integrated, multi-domain interventions. Qualitatively, the model is operationalized by defining clear roles: family physicians leading clinical screening and cognitive training, social workers organizing multi-dimensional group activities, and both parties collaborating to provide a tiered support system that effectively trains and involves family caregivers.

To overcome the identified digital divide, a pragmatic approach is recommended. This involves co-designing low-complexity, voice-assisted tools, backed by evidence from digital screeners and literacy programs, while maintaining essential non-digital services for inclusivity.

In summary, this study provides an empirical basis for shifting from fragmented, hospital-centric MCI care to a development-oriented, collaborative health governance model in the community. The proposed framework and strategies offer a practical roadmap for mitigating the public health challenge of early-stage cognitive impairment in aging societies.

## Data Availability

The raw data supporting the conclusions of this article will be made available by the authors, without undue reservation.
