# Supplementary material for: A mixed-methods study on collaborative health governance for older adults with mild cognitive impairment in Hangzhou, China
Source: Front Public Health. 2025 Nov 21;13:1720145. doi: 10.3389/fpubh.2025.1720145 (PMC12678089; doi:10.3389/fpubh.2025.1720145)
Supplement: Supplementary file 1 [file Table_1.docx]

Suppl.Table 1 Eigenvalues and Variance Contribution Rates of Factors

| Component | Eigenvalue | Variance Explained (%) | Cumulative Variance (%) |
| --- | --- | --- | --- |
| 1 | 3.791 | 18.954 | 18.954 |
| 2 | 3.495 | 17.477 | 36.431 |
| 3 | 1.692 | 8.459 | 44.89 |
| 4 | 1.363 | 6.813 | 51.703 |
| 5 | 1.242 | 6.208 | 57.911 |
| 6 | 1.102 | 5.512 | 63.422 |

Suppl.Table 2 Rotated Component Matrix

| Item | Component 1 | Component 2 | Component 3 | Component 4 | Component 5 | Component 6 |
| --- | --- | --- | --- | --- | --- | --- |
| Adaptation | 0.747 |  |  |  |  |  |
| Partnership | 0.814 |  |  |  |  |  |
| Growth | 0.681 |  |  |  |  |  |
| Affection | 0.807 |  |  |  |  |  |
| Resolve | 0.834 |  |  |  |  |  |
| Contact with family |  | 0.786 |  |  |  |  |
| Contact with friends |  | 0.807 |  |  |  |  |
| Contact with neighbors |  | 0.837 |  |  |  |  |
| Orientation to time |  |  | 0.741 |  |  |  |
| Orientation to place |  |  | 0.816 |  |  |  |
| Registration |  |  |  | 0.816 |  |  |
| Attention and calculation |  |  |  | 0.554 |  |  |
| Recall |  |  |  | 0.691 |  |  |
| Naming 1 |  |  |  |  | 0.801 |  |
| Naming 2 |  |  |  |  | 0.794 |  |
| Repetition |  |  |  |  |  | 0.525 |
| Three-stage command |  |  |  |  |  | 0.784 |
| Reading |  |  |  |  |  | 0.812 |
| Writing |  |  |  |  |  | 0.765 |
| Copying |  |  |  |  |  | 0.625 |

Suppl.Table 3 Goodness-of-Fit Indices for the Confirmatory Factor Analysis Mode

| Fit Index | Recommended Criteria | Value Obtained |
| --- | --- | --- |
| χ²/df (CMIN/DF) | 1-3 (Excellent), 3-5 (Good) | 2.197 |
| RMSEA | <0.05 (Excellent), <0.08 (Good) | 0.057 |
| Incremental Fit Index (IFI) | >0.90 (Excellent), >0.80 (Good) | 0.915 |
| Tucker-Lewis Index (TLI) | >0.90 (Excellent), >0.80 (Good) | 0.894 |
| Comparative Fit Index (CFI) | >0.90 (Excellent), >0.80 (Good) | 0.913 |

Suppl.Table 4 Comprehensive Results of the Structural Equation Modeling (SEM) Path Analysis (n=373)

| Hypothesized Path | Standardized Estimate (β) | S.E. | C.R. | p-value | 95%CI (Lower) | 95%CI (Upper) |
| --- | --- | --- | --- | --- | --- | --- |
| H1: Social Isolation → Family Support | -0.405 | 0.046 | -6.423 | <0.001 | -0.388 | -0.208 |
| H2: Family Support → Memory and Calculation | 0.176 | 0.065 | 2.396 | 0.017 | 0.028 | 0.283 |
| H3: Family Support → Reading and Praxis | -0.164 | 0.050 | -2.417 | 0.016 | -0.219 | -0.023 |
| H4: Orientation → Reading and Praxis | 0.290 | 0.116 | 2.363 | 0.018 | 0.047 | 0.501 |
| H5: Reading and Praxis → Memory and Calculation | 0.614 | 0.116 | 5.152 | <0.001 | 0.371 | 0.825 |
| H6: Memory and Calculation → Orientation | 0.400 | 0.116 | 3.304 | <0.001 | 0.158 | 0.613 |
| H7: Social Isolation → Naming Ability | 0.077 | 0.067 | 0.345 | 0.730 | -0.108 | 0.154 |
| H8: Family Support → Naming Ability | -0.058 | 0.059 | -0.339 | 0.735 | -0.136 | 0.096 |
| H9: Social Isolation → Orientation | 0.056 | 0.047 | 0.740 | 0.459 | -0.057 | 0.127 |
| H10: Social Isolation → Reading and Praxis | 0.078 | 0.042 | 1.159 | 0.246 | -0.034 | 0.131 |
| H11: Social Isolation → Memory and Calculation | -0.069 | 0.050 | -0.962 | 0.336 | -0.146 | 0.050 |
| H12: Family Support → Orientation | -0.095 | 0.058 | -1.286 | 0.198 | -0.188 | 0.039 |

Suppl.Table 5 Hypothesis Testing Results

| Hypothesis | Result |
| --- | --- |
| H1: Social isolation negatively influences the level of family support | Supported |
| H2: Family support positively influences memory and calculation ability | Supported |
| H3: Family support negatively influences reading and praxis ability | Supported |
| H4: Orientation to time and place positively influences reading and praxis ability | Supported |
| H5: Reading and praxis ability positively influences memory and calculation ability | Supported |
| H6: Memory and calculation ability positively influences orientation to time and place | Supported |
| H7: Social isolation negatively influences naming ability | Not Supported |
| H8: Family support positively influences naming ability | Not Supported |
| H9: Social isolation negatively influences orientation to time and place | Not Supported |
| H10: Social isolation negatively influences reading and praxis ability | Not Supported |
| H11: Social isolation negatively influences memory and calculation ability | Not Supported |
| H12: Family support positively influences orientation to time and place | Not Supported |

Suppl.Table 6 Basic Information on Interviewed Social Workers

| Code | Years of service | Gender | Subdistrict |
| --- | --- | --- | --- |
| A1 | 7years | female | Zhanongkou Subdistrict in Shangcheng District |
| A2 | 9years | female | Zhanongkou Subdistrict in Shangcheng District |
| A3 | 5years | female | Chengxiang Subdistrict in Xiaoshan District |
| A4 | 5years | male | Cangqian Subdistrict in Yuhang District |

Suppl.Table 7 Basic Information on Interviewed Family physician

| Code | Years of service | Gender | Subdistrict |
| --- | --- | --- | --- |
| B1 | 17years | female | Zhanongkou Subdistrict in Shangcheng District |
| B2 | 15years | female | Zhanongkou Subdistrict in Shangcheng District |
| B3 | 8years | male | Chengxiang Subdistrict in Xiaoshan District |
| B4 | 13years | male | Cangqian Subdistrict in Yuhang District |

Suppl.Table 8 Basic coding for social workers

| Open codes | Schematic of the reference points |
| --- | --- |
| Operation by a third-party center | A1: Then, when it comes to rehabilitation, it is mainly concentrated in our nursing home care center. |
| Operation by a third-party center | A1: We are not very involved in home rehabilitation. The first reason is that the houses are relatively small, and some rehabilitation equipment cannot fit through the door. What's more, we also have no elevators. |
| Perceived lack of benefit in rehabilitation for the elderly | A4: Rehabilitation? That's for kids, not for the elderly. Old people just lying in bed at home all day, none of them cares anyway. It's really something only children would do. |
| Brain games | A1: Then there are some simple handicraft activities, and some games that require a little bit of intelligence which are educational to be participated in. |
| Holiday home visits | A3: Yes, we do. During festivals such as the Double Ninth Festival or Chinese New Year, we visit people or give them gift packages. |
| Organization of recreational and cultural activities | A1: There are many, such as watching performances, which they can enjoy, and some simple handicraft activities. |
| Emotional support | For example, when it comes to psychological counseling, chatting, or similar activities, we are not particularly professional, but we will do whatever we can to help.  For example, when it comes to psychological counseling, chatting, or similar activities, we are not particularly professional, but we will try our best to help. |
| In-home daily living support services by third parties | A3: Those who are over 80 years old have a Chongyang points system, and the points can be exchanged for services such as home cleaning. |
| Meal Delivery Service | A1: For example, when delivering meals for an old people, you will definitely see him and know his condition. |
| Manual Safety Monitoring | A1: Sometimes we arrange volunteers, such as neighbors or retired workers we are familiar with, to pay attention to the elders. |
| Long-Term Care Insurance | A3: If these words refer to care, there is now a long-term care insurance that has just been launched, which can provide care services for these people. |
| Low Acceptance | A2: Yes, he doesn't know how to use it. Why would he wear something like that for no reason? He'd find it strange himself. It just doesn't make sense. |
| Limited Interregional Data Sharing | A1：See, when seniors get older, their living situation isn't always fixed. Sometimes they're here with us, other times they stay with their children for a while. If they suddenly move or are away, things can get...complicated. |
| Ease of Use | A1：The usage is relatively convenient. |
| Health Education Promotion | A3：The scope of publicity has also become broader and more convenient. |
| Improved Situational Awareness of Elders | A3：It helps us understand these individuals better and get a clearer picture of their actual situation. |
| Promotion via Official Social Media Accounts | A3：We use WeChat, WeChat official account, video channel, pretty much all of it. |
| Health-Tracking Wristband | A1：I'd say we probably focus more on the health monitoring side. You know, like making sure the elders wear their health trackers every day, that kind of thing. |
| Smart Doorbell | A1：Things like smart doorbells — we're using those a bit more now. They're internet-connected, so we can monitor everything through a dedicated platform system. |
| Low Technological Adoption among Elders | A4：But for those with cognitive impairment...They have no ability to use them, honestly. |
| Lack of Family Cooperation | A4：Some families...they don't want the community or hospitals getting involved. In those cases, we just don't follow up. |
| Family-Provided Daily Care | A3：Usually, the children live in the same neighborhood, some even in the same home. So most of the care is handled by the family, or they hire a professional caregiver. |
| Home Visits | A3：Right now, we're basically doing home visits every week. |
| Communication via WeChat | A3：We have WeChat groups, and I also add family caregivers individually |
| Weekly | A3：As for how often we talk — like I said, we're visiting weekly at this point. |
| Quarterly | A4：About once a quarter, I'd say... so four times a year. |
| Lack of Specialized Training | A4：Not really. Sometimes when they go to pick up medicine, the doctor might explain a thing or two. |
| Psychological Counseling | A3：We also provide some psychological support... that kind of thing is part of our outreach now. |
| Public Educational Lectures | A3：We're rolling out more dementia-care programs too, things like organizing family caregivers, offering basic knowledge. |
| Limited Direct Communication | A1：Actually, direct communication between us and the family physicians isn't very frequent. Our work doesn't overlap all that much. |
| Joint Home Visits | A4：The first home visit is usually done together with staff from the community health center. We have them sign a consent agreement. |
| Synchronization of Patient Status Updates | A3：Mostly it's about keeping each other updated. If there's a change in the older adult's condition, we share that. |
| Health Lectures | A1：We do invite family physicians regularly to give health lectures to seniors in the subdistrict. |
| Face-to-Face Communication | A3：And since we're located close by, sometimes I just walk over to their office to talk something through face to face. |
| Communication via WeChat | A3：We all have each other's contact information, especially WeChat. We can reach out anytime if needed. |
| Infrequent communication | A1： We don't communicate directly with family physicians very often. |
| Contact frequency was situation-dependent | A4：It's usually by phone or WeChat. We get in touch when there's a need. |

Suppl.Table 9 Initial Coding Scheme for Family Physicians

| Open Codes | Schematic of the reference points |  |
| --- | --- | --- |
| Medically-Integrated Home Care | B1：Currently, we offer two main pathways. One is our home-based medical care service, where we provide in-home medical support such as blood tests and basic health monitoring. |  |
| Assessment by Community Health Centers; Civil Affairs Services | B1：The other pathway falls under civil affairs. Based on the assessment results—which we conduct—the level of care needed is determined, and accordingly, a certain number of monthly service hours are allocated to the elderly. |  |
| LTCI Provision by Third-Party Entities | B1：Additionally, some seniors apply on their own through an online platform for Long-Term Care Insurance. |  |
| Low Community Participation | B4：The screenings are free, but participation is pretty low. Most people just don't take it very seriously. |  |
| Lack of Equipment | B2：Honestly, all we can really do here is basic screening. We don't have equipment for things like EEG or CT scans. So, that's just beyond our capacity at this point. |  |
| Lack of Targeted Health Screenings | B3：When it comes to cognitive impairment, regular health check-ups don't really cover that, as far as I know. |  |
| Implementation of Cognitive Screening | B4：We actually did run a brain health screening initiative before. We partnered with a third-party organization, the subdistrict office covered part of the cost and provided some manpower. |  |
| Suboptimal Accuracy | B3：Accuracy-wise? It's… decent. Not perfect, but definitely useful. There might be a slight margin of error, but it helps. |  |
| Ease of Use | B3： Convenience is definitely good. |  |
| High Acceptance | B3：People are generally willing to participate since it's offered free of charge. |  |
| Data Security | B2：If we're talking about our internal network—like our electronic health records and clinic system—that's all on a secure intranet. So safety and reliability aren't a concern. |  |
| Ease of Maintenance | B3：As far as I know, no maintenance is needed for now. There's a chip built into the device. |  |
| Workflow Enhancement | B3： It definitely leads to improvements. For instance, if a registered resident shows abnormal indicators, we can step in early. It makes management much more effective. |  |
| Wearable Devices | B3：Right now, we have smart wearable devices as part of our “Healthy Brain” initiative. |  |
| Electronic Health Records (EHR) | B2：But overall, our core system is still just our own electronic health records. |  |
| Inability for Independent Use | B1： And honestly, seniors with dementia… they often can't operate these devices themselves anyway. |  |
| Lack of Family Cooperation | B3：The biggest challenge? Well, for one, family caregivers aren't always cooperative. |  |
| Unrealistic Family Expectations | B1：Sometimes family caregivers have expectations that are a bit too high. The reality is, at the community level, we can only provide very basic medical care. |  |
| Time-Consuming Patient Education | B1：There are things we simply can't do. That's when it takes a lot of time and patience to communicate and explain the situation clearly to the families. |  |
| Clinician-Family Communication at Point-of-Care | B2：When residents come to our clinic for consultations, our doctors will offer guidance based on their specific needs and explain what can be done. |  |
| Enterprise WeChat | B3：We do stay in touch outside formal visits. For example, we now use our enterprise WeChat to communicate. |  |
| Shared Decision-Making for Treatment Plans | B3： Absolutely. If any intervention is needed for an elderly resident, we always seek consent from the family first. |  |
| Caregiver Training | B3： We also provide training, for example, teaching family caregivers some basic nursing skills and daily care techniques. |  |
| Push Notifications for Health Information | B1：We also push out health education content. |  |
| Delivery of Health Lectures | B1：But mostly, what we organize at the community level are regular health talks, which are general sessions. |  |
| Lack of Specialized Caregiver Training | B1: As for targeted skills training...No, that's not something we currently offer. |  |
| Psychological Counseling | B1: If we notice that a family member seems overly anxious or has other concerns, we'll refer the case to a doctor. If the doctor believes specialized intervention is needed, we help schedule an appointment at a specialist clinic. |  |
| Teleconsultation | B1: I can answer questions they might have. |  |
| Not Yet Encountered | B3: Right now, there isn't really a clear system for that. Honestly, we often end up doing things that go beyond our official responsibilities. |  |
| Synchronization of Patient Status Updates | B3: What we share mostly is basic resident information, like contact details, or when someone can't be reached, we help each other out. |  |
| Facilitating Hospital Transfers | B1： We also decide whether a home visit is necessary, or if we only help arrange a hospital transfer. |  |
| Home Visits to Special Populations | B1：There are also special visits. For example, when visiting high-risk groups, a social worker needs to accompany us. |  |
| Health Check-ups | B4：So the social workers actually organize groups and bring the elderly here for check-ups at scheduled times. |  |
| Organizing Health Lectures | B1： Say I want to organize a health talk—figuring out staffing, venue, all of that falls on us. |  |
|  |  |  |
| circumstances Context-Dependent Communication Frequency | B3：If there's an issue, we just talk it out. |  |
| Face-to-Face Communication | B3：And it's easy to meet face-to-face too, because the community center is right here, very convenient. |  |
| Communication via WeChat and Phone Calls | B3：Most of our communication happens over the phone or through WeChat. |  |
